# The Tyndall Effect in High-Resolution Computed Tomography of Semicircular Canalolithiasis with Benign Paroxysmal Positional Vertigo

**DOI:** 10.3390/diagnostics12041000

**Published:** 2022-04-15

**Authors:** Jiann-Jy Chen, Chun-Chung Lui, Tien-Yu Chen, Ping-Tao Tseng, Chao-Ming Hung

**Affiliations:** 1 Department of Otorhinolaryngology, E-Da Cancer Hospital, Kaohsiung 82445, Taiwan; jiannjy@yahoo.com.tw; 2 Prospect Clinic for Otorhinolaryngology & Neurology, Kaohsiung 81166, Taiwan; 3 Division of Medical Image, E-Da Cancer Hospital, Kaohsiung 82445, Taiwan; lchung3@gmail.com; 4 Department of Psychiatry, Tri-Service General Hospital, School of Medicine, National Defense Medical Center, Taipei 11490, Taiwan; verducciwol@gmail.com; 5 Institute of Brain Science, National Yang Ming Chiao Tung University, Taipei 11221, Taiwan; 6 Department of Psychology, College of Medical and Health Science, Asia University, Taichung 41354, Taiwan; 7 Institute of Biomedical Sciences, National Sun Yat-sen University, Kaohsiung 80424, Taiwan; 8 Division of General Surgery, Department of Surgery, E-Da Cancer Hospital, Kaohsiung 82445, Taiwan; 9 School of Medicine, College of Medicine, I-Shou University, Kaohsiung 84001, Taiwan

**Keywords:** high-resolution computed tomography, semicircular canalolithiasis, Tyndall effect, benign paroxysmal positional vertigo, clinical practice, canalithiasis

## Abstract

To date, along with the progress of new technology and computer program development, the high-resolution computed tomography (HRCT) had been applied in different clinical application, such as HRCT for coronary angiography. In the current neuroimaging reports, we present HRCT images of the head/neck of two cases, in which one had a diagnosis of benign paroxysmal positional vertigo (BPPV) and the other did not, to represent the Tyndall effect, which describes the scattering of light by particles (i.e., semicircular canalolithiasis) in the path of light and enables clinicians to see a specific signal on the HRCT images. On the HRCT image of the patient with canalolithiasis with BPPV, we could obviously see the scattering effect (i.e., Tyndall effect) in the horizontal/posterior semicircular canal; however, on the HRCT image of the other without canalolithiasis, we could not see such findings. Therefore, through the assistance of technological progress, HRCT might be beneficial in the diagnosis of semicircular canalolithiasis, which has the advantage of being noninvasive and having a low risk of complications. However, because of the disadvantages of expense and risk of radiation exposure, HRCT should be reserved for patients who are difficult to diagnose.

## 1. Introduction

The Tyndall effect, which is also known as Willis–Tyndall scattering, describes the scattering of light by particles in the path of light, which enables humans to see a beam of light [1]. The Tyndall effect has been applied to different electromagnetic waves, such as X-rays (wavelength 10 pm to 10 nm), for different clinical applications [2]. Computed tomography (CT), based on computer-processed combinations of different X-ray absorption values by different tissues from different angles, can provide 2D cross-sectional or 3D images of specific areas. The resolution of traditional CT images has been restricted by technical limitations. To date, along with the progress of new technology and computer program development, the resolution of CT has been improved. Based on the development of high-resolution CT (HRCT), clinicians should be able observe different images that could not be seen in previous traditional CT. In the current neuroimaging reports, we present HRCT images of the head/neck of two cases, in which one had a diagnosis of benign paroxysmal positional vertigo (BPPV) and the other did not, to represent the Tyndall effect on the HRCT images.

## 2. Case Reports

**Figure 1:**
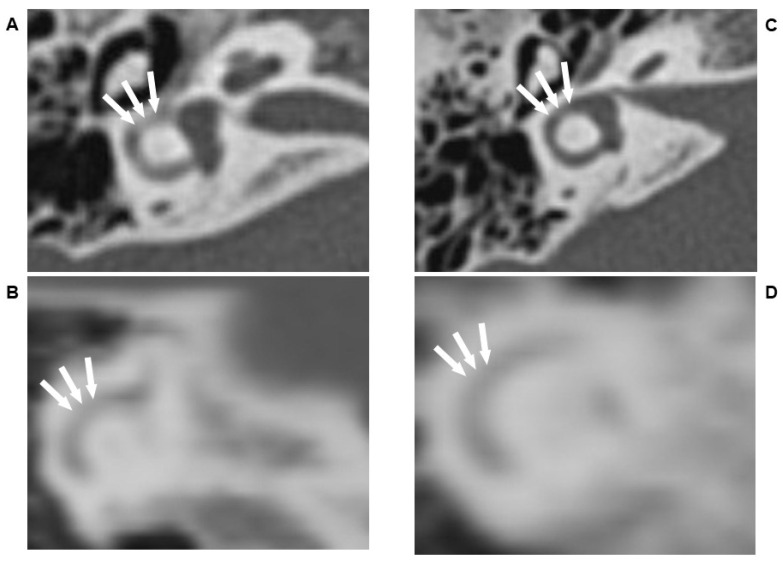
(**A**,**B**) demonstrated the horizontal semicircular canal (**A**) and posterior semicircular canal (**B**) of Ms. A, there were several snowy dots detected (white arrow), which represent the scattering effect: the X-ray had been scattered by the suspended otolith (also called otoconia, size of 10 μm) so that it could not be detected by the X-ray film and represent in white color in the final HRCT image; (**C**,**D**) demonstrated the horizontal semicircular canal (**C**) and posterioir semicircular canal (**D**) of Mrs. B, there was no any scattering effect noticed (white arrow): this was resulted from that there was no suspended otolith in the endolymph so that the X-ray could smoothly pass through the endolymph without being scattered. Therefore, after absorbed by the X-ray film, the HRCT would show black color in the final image. The detailed information for HRCT settings were: Multiplanar reformation (MPR) Dose CT dose index volume 37.42mGy FOV 270 mm Slice thickness 0.6 mm Scan mode: CT sequential CT image acquisition Partial volume can be avoided by using acquisition section width and iso voxel 0.53 × 0.53 × 0.6 mm^3^. 270/520 = 0.527.

Ms. A was a 37 y/o patient with generalized anxiety disorder with long-term insomnia. In the last 1–2 weeks, she encountered psychosocial stress, and her insomnia worsened. Along with the worsening insomnia, vertigo developed and disturbed her life, leading her to visit our clinic. The physical examination showed normal findings. The neurologic examination (NE) revealed ambiguous positive findings in the fistula test, and the right supine head to lateral test induced more prominent geotrophic nystagmus than the left test. Therefore, she was referred to the general hospital to receive a general survey. As a result, none of the surveys revealed abnormalities except for head/neck HRCT images. In the HRCT image, some snowy dots were seen among the horizontal and posterior semicircular canal, which represented the scattering effect (Figure 1A,B). The laboratory data, including complete blood count, liver function, renal function, C reactive protein, were all within normal limit. Therefore, right semicircular canalolithiasis with BPPV was diagnosed according to the Bárány Society criteria [3]. After the Barbecue maneuver for horizontal-canal BPPV and Epley maneuver for posterior-canal BPPV, the discomfort was resolved. Patient’s nystagmus disappear along with the subsided discomfort. After discussion with patient about the necessity of follow-up HRCT, the follow-up HRCT was not performed because of the patient’s fear of high radiation exposure.

Mrs. B was a 73 y/o patient with a history of tinnitus and insomnia. Since her husband’s death 1–2 years ago, her insomnia and tinnitus worsened. Transient dizziness was noticed when she could not fall asleep. There were ambiguous positive findings in the right-ear fistula test. She was referred to receive temporal bone HRCT to rule out the risk of inner ear endolymphatic fistula. In the HRCT images, there was neither a scattering effect among the horizontal/posterior semicircular canal nor other abnormalities detected (Figure 1C,D).

## 3. Discussions

**Figure 2:**
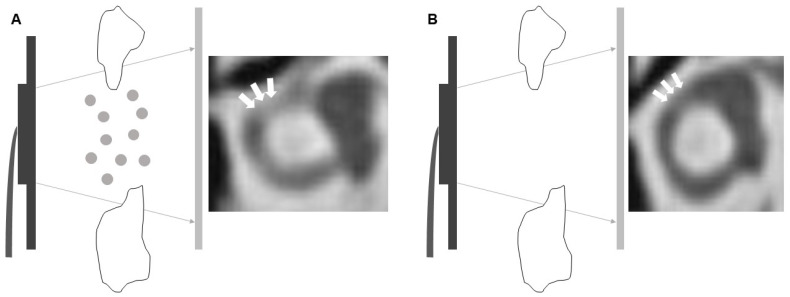
(**A**) The pathway of the X-ray beam was scattered by the suspend small particles (i.e., otolith here, size of 10 μm) so that there was scattering effect found in the final HRCT image (i.e., snowy dot) (white arrow); (**B**) The pathway of the X-ray beam smoothly go through the target organ without any scattering. Therefore, the final HRCT image showed clean and clear (i.e., black) without any snowy dot detected (white arrow).

To our knowledge, this is the first neuroimaging report providing the potential application of Tyndall effect in the clinical practice of otorhinolaryngology. The potential differential diagnosis for the neuroimage finding in Ms. A’s HRCT included fibrosis, partial volume effect, or artefact of the bony part of nearby structure. Since there is no any other evidence to approve the existence of inflammation within Ms. A’s inner ear, the possibility of fibrosis might be lower. On the other hand, the partial volume effect or artefact of the bony part of nearby structure might be a reasonable differential diagnosis in the traditional low-resolution CT, which had lower resolution. However, the initial rationale of development of HRCT was to resolve the resolution-related misdiagnosis. Therefore, although it could not be completely ruled out, the possibility partial volume effect or artefact of the bony part of nearby structure might be lower. Because of the progress of technology, the application of Tyndall effect had been used in the laboratory study to detect the suspended small particles (i.e., polyelectrolyte complexes) [4] or nanoparticles [5]. In the nano-mechanical science, the application of Tyndall effect would be helpful to transform the presentation of small invisible particles into a visible signal (i.e., scattering effect in the specific image) [6,7]. However, limited studies had been reported to address the potential application of Tyndall effect in the clinical practice. As aforementioned, the core issue of Tyndall effect was the visible scattering effect by the small suspended particles. The normally located otoliths were well-attached to their original location so that they might not result in Tyndall effect. Therefore, we might not see the Tyndall effect if the otoliths were normally attached to their original location. The current neuroimaging reports of modern HRCT, one with semicircular canalolithiasis and the other without, demonstrated the value of the Tyndall effect in the diagnosis of semicircular canalolithiasis (or so called canalithiasis). In the image of Ms. A (Figure 2A), we could see a scattering effect among the horizontal/posterior semicircular canal; in the image of Mrs. B (Figure 2B), we could not see such findings. This discrepancy resulted from the fact that, when X-rays passed through the endolymph in the horizontal/posterior semicircular canal, they were scattered by the suspended otolith (size approximately 10 μm). However, in the image of Mrs. B, who did not have semicircular canalolithiasis, the X-ray smoothly passed through the endolymph without scattering. Semicircular canalolithiasis with BPPV has a high prevalence (lifetime prevalence of 2.4%) and frequently disturbs affected patients [8]. To date, because otoliths could be observed only through microscopy, the diagnosis of semicircular canalolithiasis is mainly based on clinical symptoms and NE. However, the confirmation of semicircular canalolithiasis was not always easily by the traditional NE in patients with BPPV [9]. The misdiagnosed BPPV will be worsen after malpractice repositioning maneuver. Recently, some new CT technique had been developed to resolve this clinical difficulty. In the report by Yamane, H. et al. [10], the authors used 3-dimensional-CT (3DCT) to reconstruct the structure of semicircular canals and cupola so that the radiologist could direct see the canalolithiasis. However, the concerns of high-expensiveness and large-dose radiant exposure for the 3DCT had been a major issue. On the other hand, there was one new technique applying magnetic resonance imaging (MRI) with hydrops techniques which could help in diagnosis of BPPV. Although this new approach has a benefit of non-radiation, it is relatively more expansive and time-consuming than the HRCT. Patients with active dizziness might not be able to tolerate the long time within the MRI examination. Therefore, in comparison with the MRI, the HRCT has a more favorable cost and is less time-consuming than the MRI. Therefore, to find out other neuroimage technique with an acceptable cost and lower radiant exposure had become an emergent need of the clinicians.

## 4. Limitations

There were several potential limitations in the current study to be addressed. First, this neuroimage finding should be repeatedly confirmed in a large scale case-control trial. However, because our medical institute is a clinics, we shall not collect such a huge number of cases. Second, there was one potential concern when conducted a large number study. That is the potential radiation risk by the exposure of HRCT. Therefore, this kind of case-control study should be reserved to patients who are difficult to diagnose. Third, in the current study, we did not arrange a rechecked HRCT to confirm the repositioned otoliths because of patient’s fear of radiation overdose. In addition, the selection of controls shall be a difficult issue. Because of the concerns of radiation, it was ethically dilemma to invite patients without definite indication of HRCT to receive such a examination with high radiation.

## 5. Conclusions

The head/neck HRCT had relatively low cost and lower radiant exposure than the 3DCT of head. Through the assistance of technological progress, HRCT might be helpful in the diagnosis of semicircular canalolithiasis, which has the advantage of being noninvasive and having a low risk of complications. Based on its advantage of noninvasive, there had been studies addressing its application, although inconclusive, to detect accurate blood flow estimation [6]. Further, based on advanced technique in re-sizing gold nanoparticles, the Tyndall effect could be applied in the diagnosis and cancer treatments [11]. However, although relatively more acceptable than the 3DCT, there are still concerns about the disadvantages of expense and risk of radiation exposure. Therefore, the HRCT should be reserved for patients who are difficult to diagnose. In addition, to apply the possibility of Tyndall effect in the clinical diagnosis of BPPV would require further preclinical studies. Also, in the further preclinical studies, some other possible explanations should be ruled in and ruled out.

## Data Availability

The data of the current report was available upon reasonable request.
